# Supplementary material for: Drug-resistant focal epilepsy in children is associated with increased modal controllability of the whole brain and epileptogenic regions
Source: Commun Biol. 2022 Apr 28;5:394. doi: 10.1038/s42003-022-03342-8 (PMC9050895; doi:10.1038/s42003-022-03342-8)
Supplement: Supplementary file 2 — Supplementary Information [file 42003_2022_3342_MOESM2_ESM.pdf]

Supplementary material for manuscript:

“Drug-resistant focal epilepsy in children is associated with increased modal controllability of the whole brain and epileptogenic regions”

Supplementary Notes

Supplementary Note 1: Sensitivity analysis for group-level comparisons

(i) Repeated analysis without adjusting for age, sex and cognitive function in all 95 patients:

- Weighted Degree: No significant differences between groups, not consistent with corrected findings (control vs resective surgery cohen's d = 0.2, p = 0.58, control vs VNS cohen's d = 0.1, p = 0.85 and resective surgery vs VNS cohen's d = 0.1, p = 0.68).
- Average Controllability: Significant differences between groups, consistent with corrected findings (control vs resective surgery cohen's d =0.33, p = 0.25, control vs VNS cohen's d = 1.1, p = 3 x 10^{-4} and resective surgery vs VNS cohen's d = 0.80, p = 0.001).
- Modal Controllability: Significant differences between groups, consistent with corrected findings (control vs resective surgery cohen's d = 1.3, p = 4 x 10^{-6}, control vs VNS cohen's d = 2.0, p = 5 x 10^{-11} and resective surgery vs VNS cohen's d = 0.8, p = 9 x 10^{-4}).

(ii) Repeated analysis without adjusting for age, sex and cognitive function in only 44 patients aged >12:

- Weighted Degree: Significant differences between groups, partially consistent with corrected findings (control vs resective surgery cohen's d = 0.85, p = 0.14, control vs VNS cohen's d = 0.4, p = 0.30 and resective surgery vs VNS cohen's d = 0.4, p = 0.25).
- Average Controllability: Significant differences between groups, partially consistent with corrected findings (control vs resective surgery cohen's d = 0.3, p = 0.48, control vs VNS cohen's d = 0.8, p = 0.03 and resective surgery vs VNS cohen's d = 0.6, p = 0.14).
- Modal Controllability: Significant differences between groups, consistent with corrected findings (control vs resective surgery cohen's d = 1.0, p = 0.005, control vs VNS cohen's d 1.4, p = 2 x 10^{-4} and resective surgery vs VNS cohen's d = 0.4, p = 0.30).

Supplementary Tables

**Atlas Parcel**	**Terminologia Anatomica Nuclei**	**Function**
anterior	Anterior	Output to cingulate/parahippocampal gyri; common target for DBS for epilepsy
central lateral, lateral posterior, medial pulvinar	CL & CM	Output to motor cortex and striatum; common target for DBS for epilepsy
ventral latero-ventral	VL & VPL	Motor (VL) and sensory/insula (VPL) projections; common targets for DBS for dystonia (VL) and pain (VPL)
ventral latero-ventral	LP	
medio-dorsal	MD	Output to prefrontal cortex; targets for DBS for obsessive-compulsive disorder

Supplementary Table 1: Atlas-based thalamic nuclei which have increased modal controllability, their corresponding terminologia anatomica nuclei and function. CL = central latera, CM = centromedian, VL = ventral lateral, VPL = ventral posterolateral, LP = lateral posterior, MD = medial dorsal.

Supplementary Figures

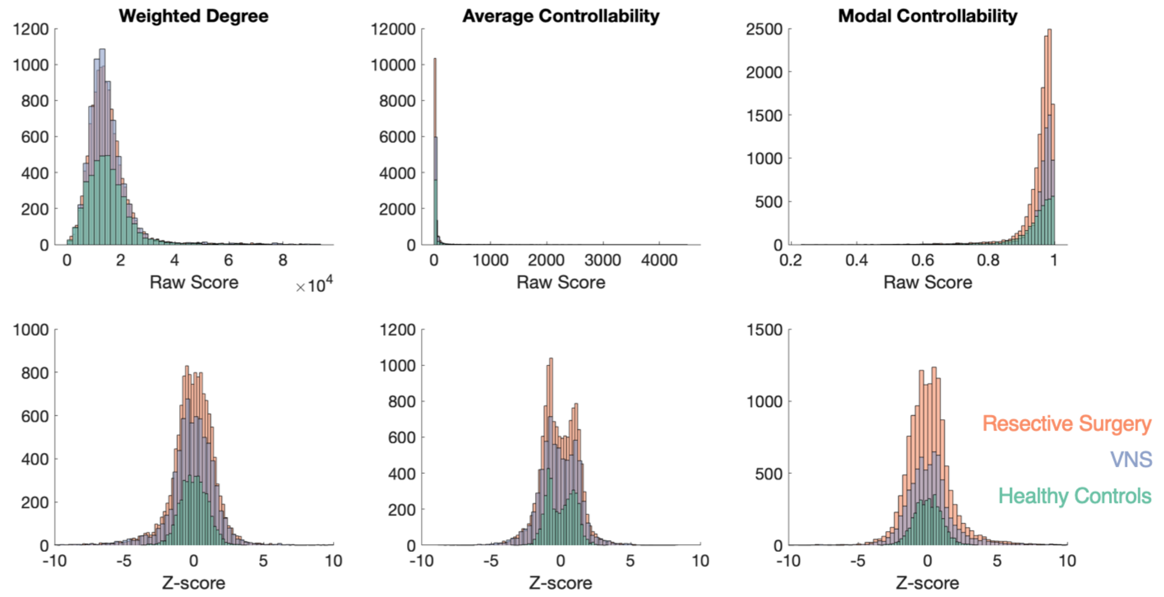

Supplementary Figure 1: Histograms illustrating the non-parametric nature of the raw scores (top panel) which were normalised by converting each value to a rank within each individual patient and using the mean and standard deviation of the ranks in the healthy controls to calculate a Z-score for each parcel (bottom panel).

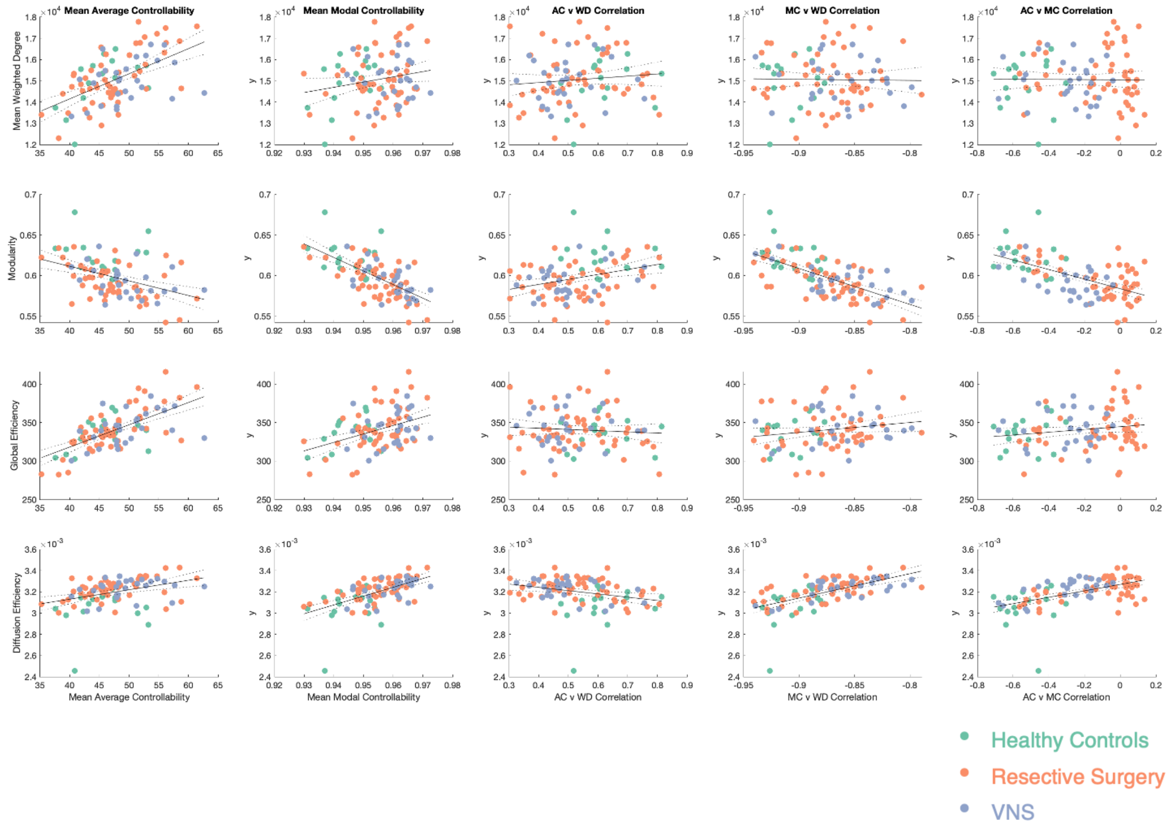

Supplementary Figure 2: Relationships between five controllability variables and four traditional graph theory metrics for each of the patients. AC = average controllability, MC = modal controllability and WD = weighted degree. Lines show correlation coefficients between measures +/- 95% confidence intervals. Note that the groups seem to segregate best by AC vs MC correlation and none of the graph metrics correlate strongly with this measure.

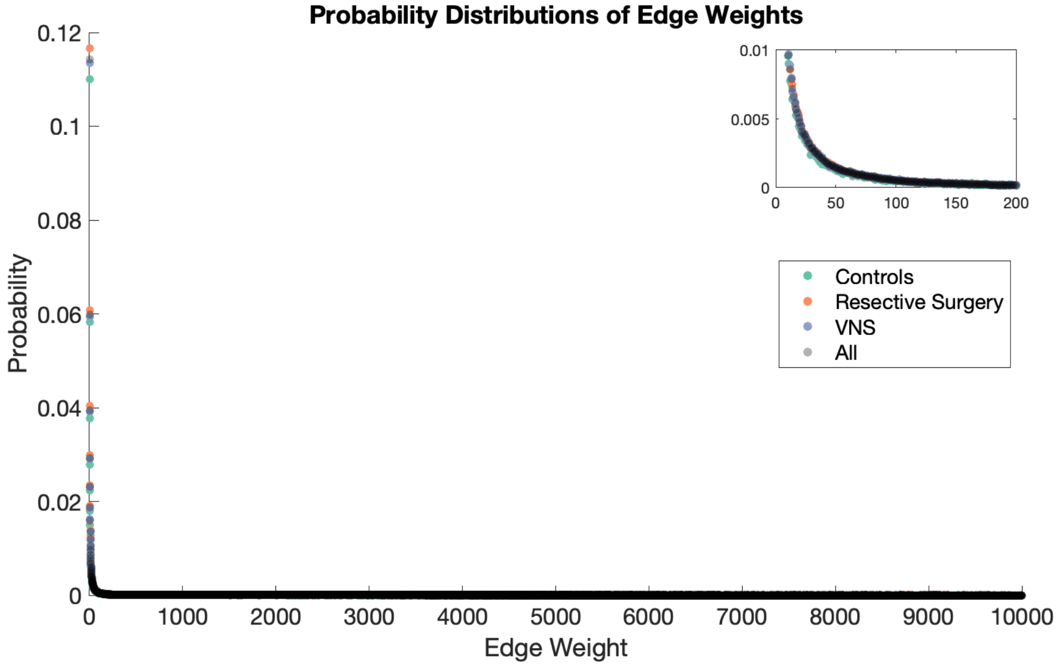

Supplementary Figure 3: Probability distributions of the edge weights from the 3 groups of patients and all groups combined (in grey), showing similar distributions across the groups. Inset shows same graph zoomed in showing strong overlap between groups. The overall distribution curve was best fit using Weibull distribution with a = 0.717 and b = 0.325 (RMS of error 2.3 x 10^{-5}), providing a better fit than an exponential distribution (RMS of error 3.4 x 10^{-4}). Curves were fit using the curve fitting toolbox in matlab.

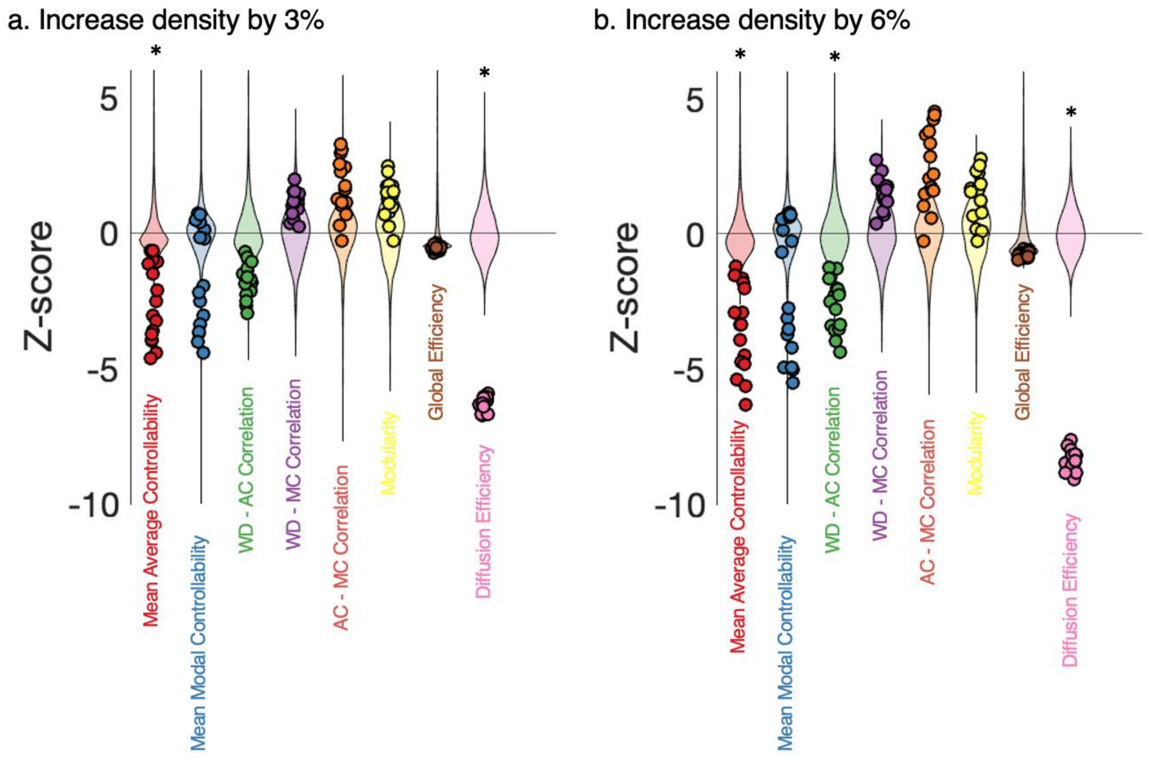

Supplementary Figure 4: Increasing the density of connections in healthy controls at random does not explain the changes observed in children with drug-resistant epileps. *Z-scores of graph theory and controllability metrics of the original connectomes from the healthy controls compared to 3200 simulations (200 for each subject, shown as background violin plots) for each subject where extra edges were added. Adding edges (3% = 1000 edges, 6% = 2000 edges) leads to a statistically significant increase in average controllability and diffusion efficiency, and a trend towards increase in modal controllability and decrease in modularity. This does not recapitulate the changes seen in the patients with epilepsy. The WD-AC correlation significantly increases, WC-MC and AC-MC correlations do not change significantly, indicating that the 'disorga. * indicates that average Z-score of the controls (n=16) compared to Z-scores from all the simulations (n=3200) is > 2.5 (effectively correcting for 8 multiple comparisons).*

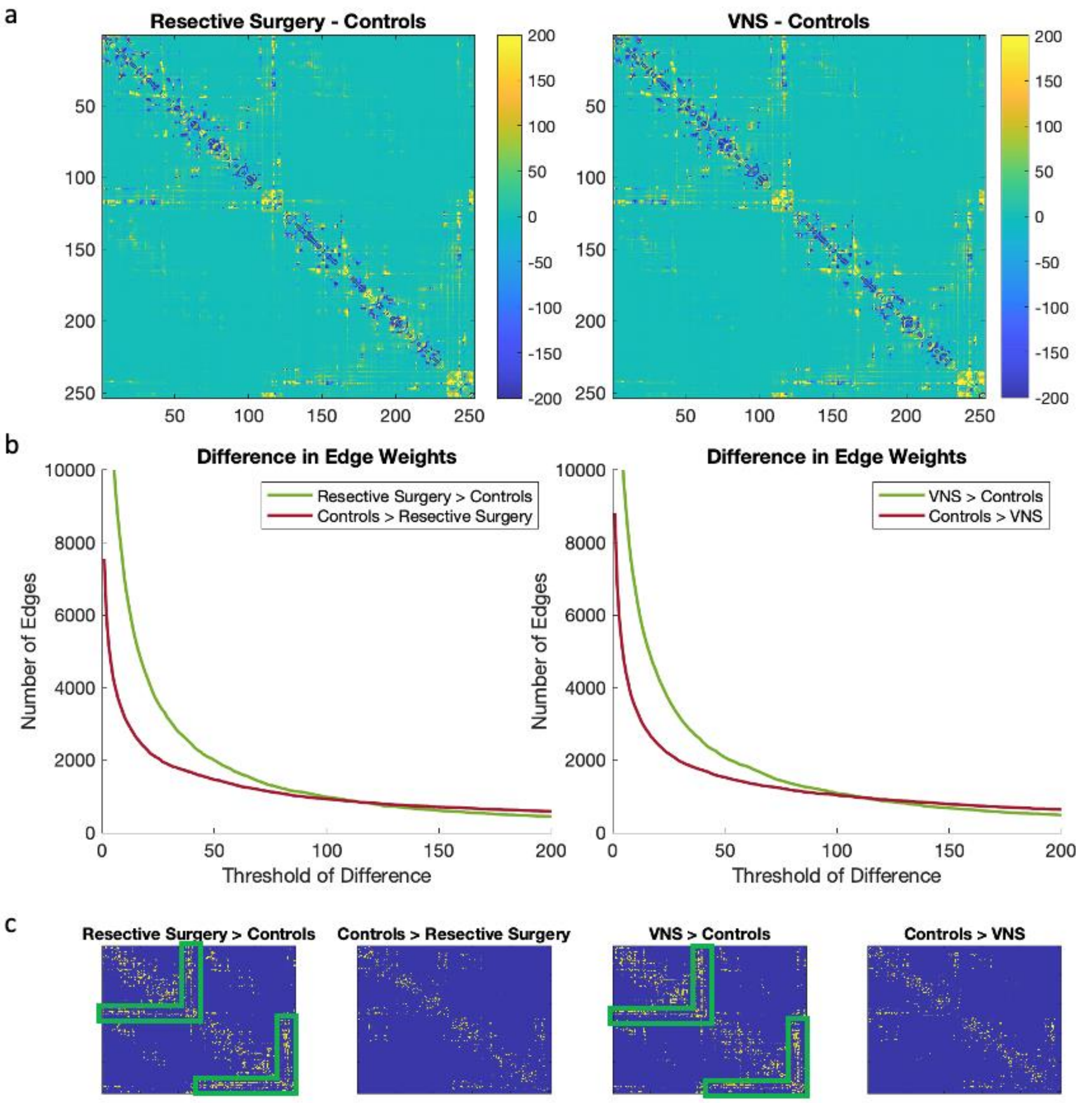

Supplementary Figure 5: Average structural connectivity matrix differences between controls and patients with drug-resistant epilepsy. *(a) Average structural connectivity matrices for each group were constructed and the differences between resective surgery and controls (left) and VNS and controls (right) are shown. Colour maps display the number of streamlines. (b) Quantification of the number of edges different between controls and patients above a certain threshold. There were more low edge weight differences (>100 streamlines) in the patients and high weight edge differences (>100 streamlines) in the controls. (c) Location of these low edge weight difference connections (20-80 streamlines) in the structural connectivity matrices reveals that additional connections in patients were predominantly in ipsilateral thalamocortical connections (green boxes) with other scattered ipsilateral cortico-cortical connections having both increased and decreased weight in the patients compared to controls.*

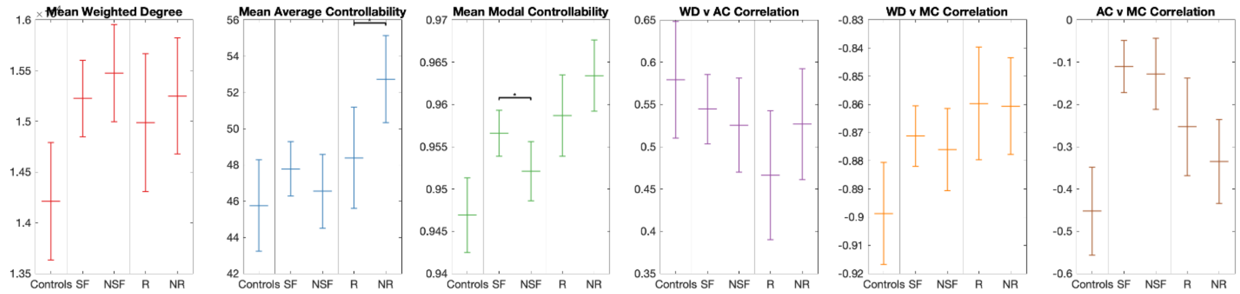

Supplementary Figure 6: Global controllability do not associate with treatment outcome. *Estimated marginal means (+/- 95% Wald confidence interval) for the different groups, stratified by treatment outcomes. Following adjustment for age, sex, cognitive function and mean weighted degree, pairwise comparisons (adjusted for multiple comparisons) between groups resulted in there being small differences between the VNS responders and non-responders for mean average controllability (p=0.02) and seizure-free and non seizure-free resective surgery patients for mean modal controllability (p=0.05).*

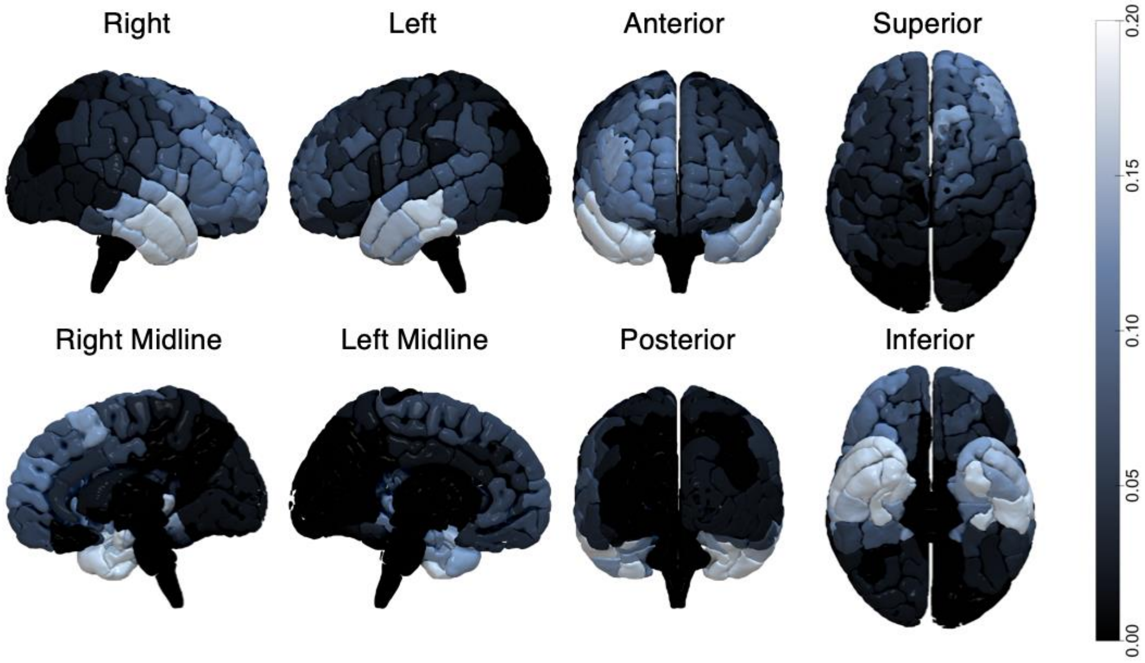

Supplementary Figure 7: Composite figure of resected parcels across 52 patients. *Views of a template paediatric brain showing the proportion of parcels that were resected across the 52 resective surgery patients. Colour maps display the % of patients that have had that parcel resected. As would be expected, the most commonly resected parcels were the anterior and mesial temporal lobe structures (right > left) with fewer resections involving midline and posterior parcels.*

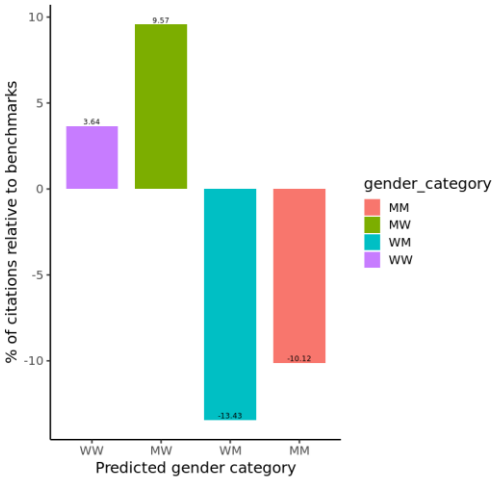

Supplementary Figure 8: Citation diversity. *Percentage of citations above/below threshold from leading neuroscience journals of different predicted gender categories (M = man and W = woman)*
