# Supplementary material for: Drug-resistant focal epilepsy in children is associated with increased modal controllability of the whole brain and epileptogenic regions
Source: Commun Biol. 2022 Apr 28;5:394. doi: 10.1038/s42003-022-03342-8 (PMC9050895; doi:10.1038/s42003-022-03342-8)
Supplement: Supplementary file 3 — Description of Additional Supplementary Files [file 42003_2022_3342_MOESM3_ESM.pdf]

Description of Additional Supplementary Files

File name: Supplementary Data 1

Description: Group and demographic details of each participant. Cognitive class categories are 1 = >2SD below the mean, 2 = 1-2SD below the mean, 3 = 0-1 SD below the mean, 4 = 0-1 SD above the mean, N/A = not assessed. For VNS, response = >50% reduction in seizures. SF = seizure free, NSF = not seizure free, MCD = malformation of cortical development, FCD = focal cortical dysplasia.
