# Supplementary material for: Drug-resistant focal epilepsy in children is associated with increased modal controllability of the whole brain and epileptogenic regions
Source: Commun Biol. 2022 Apr 28;5:394. doi: 10.1038/s42003-022-03342-8 (PMC9050895; doi:10.1038/s42003-022-03342-8)
Supplement: Supplementary file 6 — Reporting Summary [file 42003_2022_3342_MOESM6_ESM.pdf]

nature portfolio

Corresponding author(s): Rod C Scott

Last updated by author(s): Jan 12, 2022

Reporting Summary

Nature Portfolio wishes to improve the reproducibility of the work that we publish. This form provides structure for consistency and transparency in reporting. For further information on Nature Portfolio policies, see our Editorial Policies and the Editorial Policy Checklist.

Statistics

For all statistical analyses, confirm that the following items are present in the figure legend, table legend, main text, or Methods section.

n/a	Confirmed	
☐	☒	The exact sample size (n) for each experimental group/condition, given as a discrete number and unit of measurement
☐	☒	A statement on whether measurements were taken from distinct samples or whether the same sample was measured repeatedly
☐	☒	The statistical test(s) used AND whether they are one- or two-sided<br>*Only common tests should be described solely by name; describe more complex techniques in the Methods section.*
☐	☒	A description of all covariates tested
☐	☒	A description of any assumptions or corrections, such as tests of normality and adjustment for multiple comparisons
☐	☒	A full description of the statistical parameters including central tendency (e.g. means) or other basic estimates (e.g. regression coefficient) AND variation (e.g. standard deviation) or associated estimates of uncertainty (e.g. confidence intervals)
☐	☒	For null hypothesis testing, the test statistic (e.g. F, t, r) with confidence intervals, effect sizes, degrees of freedom and P value noted<br>*Give P values as exact values whenever suitable.*
☒	☐	For Bayesian analysis, information on the choice of priors and Markov chain Monte Carlo settings
☒	☐	For hierarchical and complex designs, identification of the appropriate level for tests and full reporting of outcomes
☐	☒	Estimates of effect sizes (e.g. Cohen's d, Pearson's r), indicating how they were calculated

Our web collection on statistics for biologists contains articles on many of the points above.

Software and code

Policy information about availability of computer code

Data collection	N/A
Data analysis	Matlab v2020<br>MR Trix<br>Code available at https://github.com/aswinchari/NetworkControl/

For manuscripts utilizing custom algorithms or software that are central to the research but not yet described in published literature, software must be made available to editors and reviewers. We strongly encourage code deposition in a community repository (e.g. GitHub). See the Nature Portfolio guidelines for submitting code & software for further information.

Data

Policy information about availability of data

All manuscripts must include a data availability statement. This statement should provide the following information, where applicable:

- Accession codes, unique identifiers, or web links for publicly available datasets
- A description of any restrictions on data availability
- For clinical datasets or third party data, please ensure that the statement adheres to our policy

All code and structural connectivity matrices are available on github (www.github.com/aswinchari/NetworkControl).

Field-specific reporting

Please select the one below that is the best fit for your research. If you are not sure, read the appropriate sections before making your selection.

- [x] Life sciences
- [ ] Behavioural & social sciences
- [ ] Ecological, evolutionary & environmental sciences

For a reference copy of the document with all sections, see nature.com/documents/nr-reporting-summary-flat.pdf

Life sciences study design

All studies must disclose on these points even when the disclosure is negative.

Sample size	No sample size calculations. All eligible patients were identified in relevant time period undergoing state-of-the-art diffusion sequences from 2015 to 2020.
Data exclusions	As stated in methods, children aged<3 were excluded. In addition, 3 resective surgery patients and 1 control were excluded due to parcellation errors.
Replication	N/A
Randomization	N/A
Blinding	N/A

Reporting for specific materials, systems and methods

We require information from authors about some types of materials, experimental systems and methods used in many studies. Here, indicate whether each material, system or method listed is relevant to your study. If you are not sure if a list item applies to your research, read the appropriate section before selecting a response.

Materials & experimental systems

n/a	Involved in the study	
x		Antibodies
x		Eukaryotic cell lines
x		Palaeontology and archaeology
x		Animals and other organisms
	x	Human research participants
	x	Clinical data
x		Dual use research of concern

Methods

n/a	Involved in the study	
x		ChIP-seq
x		Flow cytometry
	x	MRI-based neuroimaging

Human research participants

Policy information about studies involving human research participants

Population characteristics	All children with drug-resistant epilepsy or controls.
Recruitment	Retrospective recruitment based on those undergoing surgery or VNS insertion
Ethics oversight	Joint Research Office of Great Ormond Street Hospital & University College London Institute of Child Health (project ID 19BI26).

Note that full information on the approval of the study protocol must also be provided in the manuscript.

Clinical data

Policy information about clinical studies

All manuscripts should comply with the ICMJEguidelines for publication of clinical research and a completedCONSORT checklist must be included with all submissions.

Clinical trial registration	N/A as retrospective study. Protocol was approved by Joint Research Office of Great Ormond Street Hospital & University College London Institute of Child Health (project ID 19BI26).
Study protocol	Not a clinical trial.
Data collection	2015-2020. All from single centre

Outcomes: MRI data and seizure freedom

Magnetic resonance imaging

Experimental design

Design type: Diffusion imaging, resting state

Design specifications: N/A

Behavioral performance measures: N/A. Outcomes were surrounding network measures and seizure freedom following surgery

Acquisition

Imaging type(s): Diffusion

Field strength: 3T

Sequence & imaging parameters: The protocol included a T1 MPRAGE sequence and multi-shell diffusion sequence employing a diffusion-weighted spin-echo single shot 2D EPI acquisition, with multi-band radio frequency pulses to accelerate volume coverage along the slice direction. A multi-band factor of 2 was used to image 66 slices of 2 mm thickness with 0.2 mm slice gap. Diffusion gradients were applied over two 'shells': b = 1000 s/mm2 and b = 2200 s/mm2, with 60 non-collinear diffusion directions per shell in addition to 13 interleaved b = 0 (non-diffusion weighted) images. Other imaging parameters were: TR = 3050 ms, TE = 60 ms, field of view = 220 mm × 220 mm, matrix size = 110 × 110, in-plane voxel resolution = 2.0 mm × 2.0 mm, GRAPPA factor 2, phase-encoding (PE) partial Fourier = 6/8. An additional b = 0 scam was acquired, with identical readout to the diffusion-weighted scan, but with the phase encode direction flipped by 180° (in the anterior-posterior direction), for correction of susceptibility-related artifacts.

Area of acquisition: Whole brain

Diffusion MRI: [x] Used [ ] Not used

Parameters: The protocol included a T1 MPRAGE sequence and multi-shell diffusion sequence employing a diffusion-weighted spin-echo single shot 2D EPI acquisition, with multi-band radio frequency pulses to accelerate volume coverage along the slice direction. A multi-band factor of 2 was used to image 66 slices of 2 mm thickness with 0.2 mm slice gap. Diffusion gradients were applied over two 'shells': b = 1000 s/mm2 and b = 2200 s/mm2, with 60 non-collinear diffusion directions per shell in addition to 13 interleaved b = 0 (non-diffusion weighted) images. Other imaging parameters were: TR = 3050 ms, TE = 60 ms, field of view = 220 mm × 220 mm, matrix size = 110 × 110, in-plane voxel resolution = 2.0 mm × 2.0 mm, GRAPPA factor 2, phase-encoding (PE) partial Fourier = 6/8. An additional b = 0 scam was acquired, with identical readout to the diffusion-weighted scan, but with the phase encode direction flipped by 180° (in the anterior-posterior direction), for correction of susceptibility-related artifacts.

Preprocessing

Preprocessing software: MRTRIX

Normalization: Not normalised - in patient space

Normalization template: N/A

Noise and artifact removal: Following preprocessing (denoising, eddy correction and bias correction), response was estimated using multi-shell, multi-tissue constrained spherical deconvolution that exploits the unique b-value dependencies of different tissue types and produces more accurate apparent fibre density measures

Volume censoring: N/A

Statistical modeling & inference

Model type and settings: GLM

Effect(s) tested: N/A

Specify type of analysis: [ ] Whole brain [ ] ROI-based [x] Both

Anatomical location(s): 253 locations based on atlas

Statistic type for inference (See Eklund et al. 2016): Network analysis

Correction: Benjamini-hochberg + use of conservative Z-score thresholds

Models & analysis

n/a	Involved in the study
☒	☐ Functional and/or effective connectivity
☐	☒ Graph analysis
☒	☐ Multivariate modeling or predictive analysis

Graph analysis	Controllability was the main graph analysis, based on the paper by Shi Gu: https://www.nature.com/articles/ncomms9414
